# Decreased neuroinflammation correlates to higher vagus nerve activity fluctuations in near-term ovine fetuses: a case for the afferent cholinergic anti-inflammatory pathway?

**DOI:** 10.1186/s12974-016-0567-x

**Published:** 2016-05-10

**Authors:** M. G. Frasch, M. Szynkaruk, A. P. Prout, K. Nygard, M. Cao, R. Veldhuizen, R. Hammond, B. S. Richardson

**Affiliations:** Department of Obstetrics and Gynaecology, CHU Ste-Justine Research Centre, Faculty of Medicine, Université de Montréal, Montréal, QC Canada; Department of Neurosciences, CHU Ste-Justine Research Centre, Faculty of Medicine, Université de Montréal, Montréal, QC Canada; Animal Reproduction Research Centre (CRRA), Faculty of Veterinary Medicine, Université de Montréal, Montréal, QC Canada; Department of Obstetrics and Gynaecology, Lawson Health Research Institute, University of Western Ontario, London, ON Canada; Microscopy Imaging@Biotron, University of Western Ontario, London, ON Canada; Department of Physiology and Pharmacology, University of Western Ontario, London, ON Canada; Department of Pathology, University of Western Ontario, London, ON Canada; Department of Obstetrics and Gynecology, University of Washington, 1959 NE Pacific St, Box 356460, Seattle, WA 98195 USA

**Keywords:** Fetus, Labor, Vagus, Microglia, HMGB1, CHRNA7, HRV, RMSSD

## Abstract

**Background:**

Neuroinflammation in utero may contribute to brain injury resulting in life-long neurological disabilities. The pivotal role of the efferent cholinergic anti-inflammatory pathway (CAP) in controlling inflammation, e.g., by inhibiting the HMGB1 release, via the macrophages’ α7 nicotinic acetylcholine receptor (α7nAChR) has been described in adults, but its importance in the fetus is unknown. Moreover, it is unknown whether CAP may also exert anti-inflammatory effects on the brain via the anatomically predominant afferent component of the vagus nerve.

**Methods:**

We measured microglial activation in the ovine fetal brain near term 24 h after the umbilical cord occlusions mimicking human labor versus controls (no occlusions) by quantifying HMGB1 nucleus-to-cytosol translocation in the Iba1+ and α7nAChR+ microglia. Based on multiple clinical studies in adults and our own work in fetal autonomic nervous system, we gauged the degree of CAP activity in vivo using heart rate variability measure RMSSD that reflects fluctuations in vagus nerve activity.

**Results:**

RMSSD correlated to corresponding plasma IL-1β levels at *R* = 0.57 (*p* = 0.02, *n* = 17) and to white matter microglia cell counts at *R* = −0.89 (*p* = 0.03). The insult increased the HMGB1 translocation in α7nAChR+ microglia in a brain region-dependent manner (*p* < 0.001). In parallel, RMSSD at 1 h post insult correlated with cytosolic HMGB1 of thalamic microglia (*R* = −0.94, *p* = 0.005), and RMSSD at pH nadir correlated with microglial α7nAChR in the white matter (*R* = 0.83, *p* = 0.04). Overall, higher RMSSD values correlated with lower HMGB1 translocation and higher α7nAChR intensity per area in a brain region-specific manner.

**Conclusions:**

Afferent fetal CAP may translate increased vagal cholinergic signaling into suppression of cerebral inflammation in response to near-term hypoxic acidemia as might occur during labor. Our findings suggest a new control mechanism of fetal neuroinflammation via the vagus nerve, providing novel possibilities for its non-invasive monitoring in utero and for targeted treatment.

**Electronic supplementary material:**

The online version of this article (doi:10.1186/s12974-016-0567-x) contains supplementary material, which is available to authorized users.

## Background

Induced animal sepsis and clinical-pathologic studies in adults indicate that loss of the cholinergic anti-inflammatory pathway’s (CAP) inhibitory influence unleashes innate immunity, producing higher levels of pro-inflammatory mediators that exacerbate tissue damage. This decrease in CAP activity also decreases short-term heart rate variability (HRV), e.g., as measured by the beat-to-beat HRV measures, such as root mean square of successive differences in R-R intervals of ECG (RMSSD), a measure of vagal modulation of HRV [1, 2]. Thus, short-term HRV measures reflect CAP activity in adults [3]. Of note, RMSSD also reflects vagal activity in fetal sheep [4].

Increased CAP vagal activity inhibits the release of pro-inflammatory cytokines such as interleukin (IL)-1β [1]. This systemic CAP effect is mediated via the α7 nicotinic acetylcholine receptor (α7nAChR) expressed on macrophages [5]. However, recent studies have shown a similar α7nAChR-dependent effect in brain microglia in vitro [6–8].

In adult species, high-mobility group box protein 1 (HMGB1), a non-histone DNA-binding protein, acts as an important pro-inflammatory cytokine linking necrosis with ensuing inflammation by translocating from the neuronal nucleus to the cytosol and then to the extracellular space, leading to microglial activation [9]. Much attention has been paid to the effects of α7nAChR stimulation on HMGB1 secretion because of its therapeutic potential to treat sepsis; HMGB1 represents a crucial link between neuronal necrosis and the cerebral inflammatory response mediated by microglia, thus impacting the long-term outcome of neurological injury [9, 10]. HMGB1 also acts as a potent pro-inflammatory cytokine when secreted by microglia in response to inflammatory stimuli [11]. This requires translocation of HMGB1 from nucleus to cytosol [9].

Systemic and neuroinflammation have been implicated as important pathophysiological mechanisms acting independently to cause fetal brain injury or contributing to hypoxic-asphyxial brain injury with consequences for postnatal health [12, 13]. In the late-gestation ovine and human fetus, the autonomic nervous system and cholinergic vagal activity in particular are known to be sufficiently mature [2, 14].

We have shown that CAP is active spontaneously near term, such that individual baseline RMSSD values and the levels of the pro-inflammatory cytokines IL-1β and IL-6 are inversely correlated, reflecting spontaneous CAP activity [15].

First, we hypothesized that the fetal inflammatory response induced by hypoxic acidemia will result in an increase of systemic CAP activity as a compensatory mechanism and an inhibitory effect of CAP on the cerebral inflammatory response. The systemic inflammatory response will be reflected by an increased vagal activity and hence a correlation of RMSSD and IL-1β.

Second, we sought to determine the effect of fetal hypoxic-acidemia insult on brain regional activation of the microglia expressing α7nAChR, and the relation of systemic and cerebral CAP activation to the intracellular HMGB1 localization in these cells. Thus, we hypothesized that the cerebral inflammatory response will result in microglial HMGB1 translocation from the nucleus to the cytosol due to increased microglial activation via α7nAChR and this HMGB1 translocation will correlate with the degree of CAP’s vagal activation measured by RMSSD.

## Results

As reported, repetitive umbilical cord occlusions (UCO) resulted in worsening acidosis over 3 to 4 h and eventually a severe degree of acidemia, fetal pH 7.36 ± 0.03 to 6.90 ± 0.13 (*p* < 0.01) [16].

**Fig. 1:**
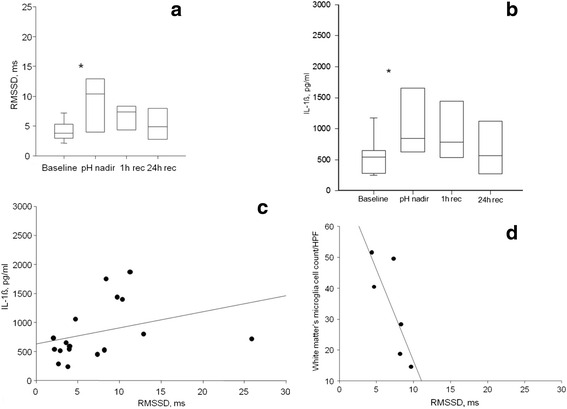
**a**, **b** Fetal inflammatory response to acute hypoxic acidemia. *p < 0.05 versus baseline. pH nadir, at pH <7.00; recovery 1 and 24 h after the pH nadir. Median ± {5–95 %}. **c** IL-1β measured at baseline, pH nadir, and 1 h or recovery correlates to RMSSD (*R* = 0.57, *p* = 0.02, *n* = 17). Baseline values were chosen for lactate >1.5 mmol/l. **d** White matter microglia cell counts at 24 h recovery correlate to RMSSD at 1 h of recovery (*R* = 0.89, *p* = 0.03, *n* = 6)

RMSSD and IL-1β increased ~2-fold from baseline versus the time of nadir pH (*p* < 0.05) and fell by 1 h of recovery (Fig. 1a, b; for IL-1β cf. [17]). Of note, at 1 h of recovery, the values of RMSSD and IL-1β were still clearly, but not statistically significantly elevated. Baseline, nadir pH, and 1 h of recovery RMSSD correlated to corresponding IL-1β levels at *R* = 0.57 (*p* = 0.02, *n* = 17, Fig. 1c).

**Fig. 2:**
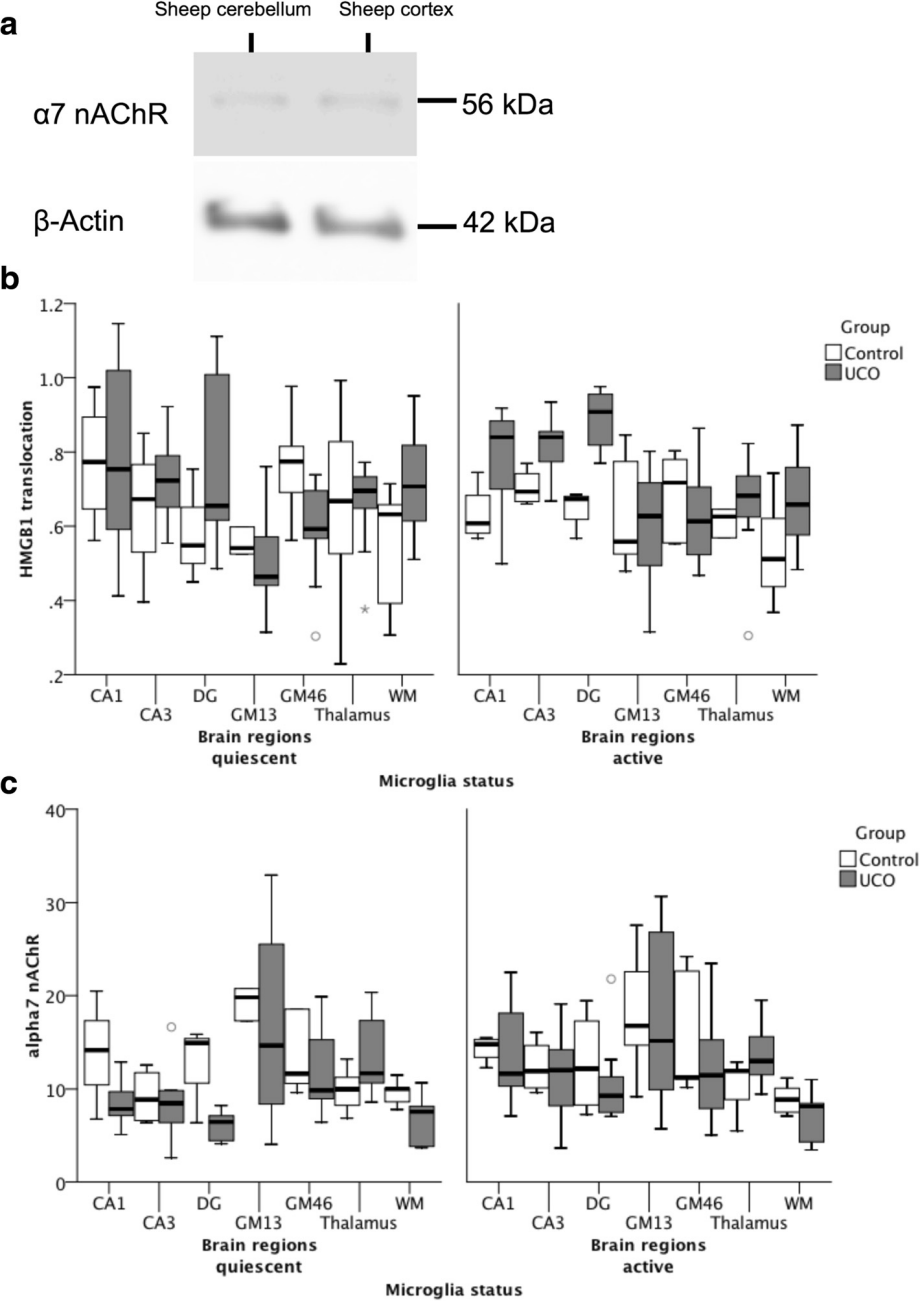
**a** Presence of α7nAChR in near-term ovine brain was confirmed by Western blot. **b** Effect of UCO, microglia status and brain regions on HMGB1 translocation: significant group*brain region*microglia status interaction (*p* < 0.001). See Additional file 1: Table S1 for details. **c** Effect of UCO and microglia status on α7 nAChR immunofluorescence measured as intensity per area: significant group*microglia status*HMGB1 translocation interaction (*p* < 0.001). See Additional file 1: Table S2 for details. Note that, for α7 nAChR signal, between-brain region comparisons were not possible, because gain settings were optimized for each brain region and kept constant between cell compartment and groups (but not from region to region). HMGB1 signal is expressed as ratio of cytosolic to nuclear signal, i.e., the higher the ratio, the more HMGB1 translocation is observed; this normalization permits between-brain region comparisons

Fetal gender did not contribute to this correlation. As reported, MG cell counts were increased in the white matter of the treated animals versus the control group [17]. RMSSD measurements at baseline and 1 h of recovery correlated inversely to white matter MG cell counts determined at 24 h of recovery at *R* = −0.71 (*p* = 0.05) and *R* = −0.89 (*p* = 0.03), respectively (Fig. 1d).

We confirmed the specificity of the α7nAChR antibody for ovine brain tissue and the presence of the receptor in the ovine cerebellum and cortex (Fig. 2).

**Fig. 3:**
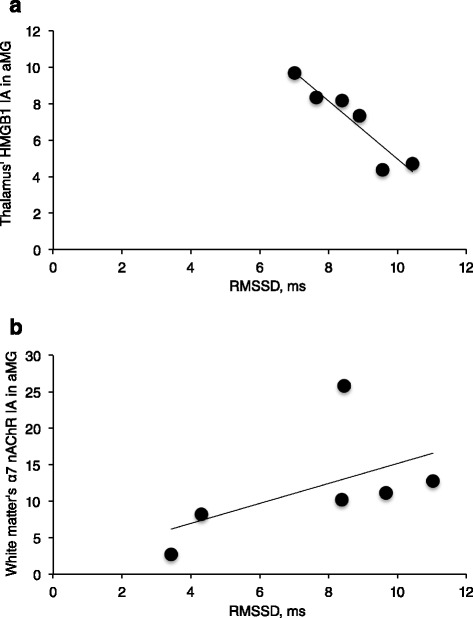
UCO group: HMGB1 and α7 nAChR immunofluorescence are shown, measured as intensities per area (I/A) in activated microglia (aMG) in relation to RMSSD as marker of CAP activity. RMSSD at 1 h recovery negatively correlates with cytoplasmic HMGB1 in thalamus **a** *R* = −0.94, *p* = 0.005. RMSSD at pH nadir positively correlates with α7 nAChR in the white matter **b** *R* = 0.83, *p* = 0.04

For both HMGB1 translocation index and α7nAChR expression, the main effect of belonging to the UCO or the control group was not significant (*p* = 0.25 and *p* = 0.54, respectively, Fig. 2). However, the effect of group on HMGB1 translocation differed according to brain reg (mostly in hippocampus and GM46) and microglia status (*p* < 0.001 for interaction terms) (Additional file 1: Table S2). Interestingly, statistically significant interaction effects on HMGB1 translocation were observed in cortical and hippocampal regions, but not in the subcortical (thalamus, white matter) brain regions. In gray matter, these effects applied to quiescent MG (qMG), but not to activated MG (aMG); meanwhile, in the hippocampus, this was mostly apparent in aMG.

Similarly, a model that accounted for interactions of group and microglia status and HMGB1 was able to predict α7nAChR expression (*p* < 0.001). Notably, the effect of UCO group was in the same direction but of much greater magnitude in aMG compared to qMG (Additional file 1: Table S3).

In parallel, RMSSD at 1 h of recovery correlated highly with cytosolic HMGB1 intensity per area in aMG of thalamus (*R* = −0.94, *p* = 0.005, Fig. 3a) and RMSSD at pH nadir correlated with α7nAChR intensity per area in aMG of WM (*R* = 0.83, *p* = 0.04, Fig. 3b). Similar to the relationship shown in Fig. 3a, within both qMG and aMG in WM, HMGB1 translocation index correlated to RMSSD at 1 h of recovery (*R* = −0.83, *p* = 0.04 and *R* = −0.89, *p* = 0.02, respectively). This finding was again replicated for qMG of GM13 and aMG of GM46: HMGB1 translocation index correlated there to RMSSD at pH nadir (*R* = −0.99, *p* < 0.001 and *R* = −0.83, *p* = 0.04, respectively). That is, higher RMSSD values correlated with lower HMGB1 translocation and higher α7nAChR intensity per area in brain region-specific and microglia status-specific manner.

**Fig. 4:**
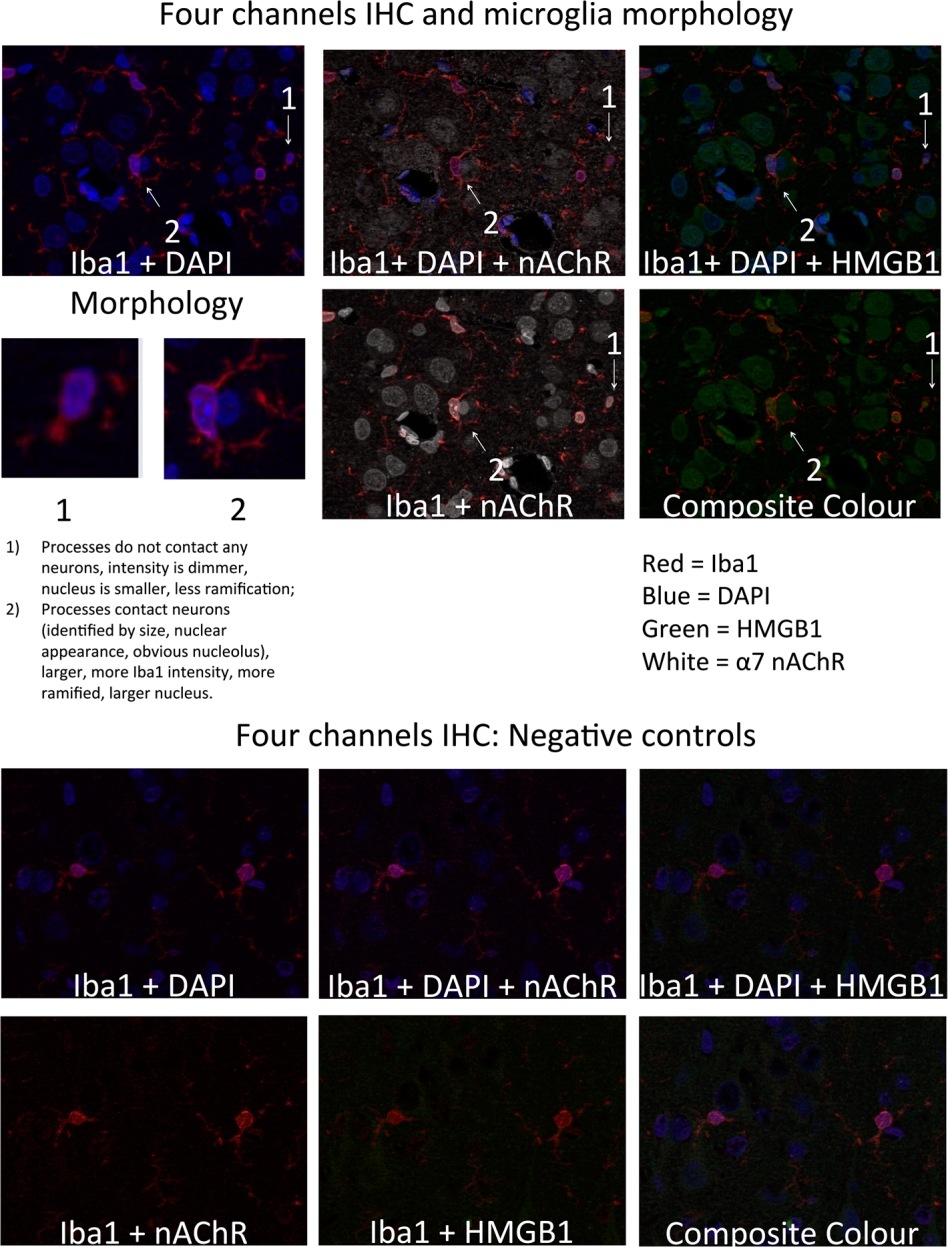
α7nAChR + HMGB1 + Iba1 + DAPI immunofluorescent staining: *Red* = Iba1; *Blue* = DAPI; *Green* = HMGB1; *White* = α7nAChR. Images were made from extended depth of focus, maximum intensity projections from convexial gray matter layers 4 to 6. *Top*: Features of active versus inactive microglia: Inactive cells have no interaction with neurons, are smaller, less bright, and less ramified versus the activated cells. *Bottom*: Co-localization staining for Iba1+ cells expressing α7nAChR with intracellular localization of HMGB1 and DAPI counterstain. **a** Iba1 + DAPI + α7nAChR; **b** Iba1 + DAPI + HMGB1; **c** Iba1+ α7nAChR; **d** best focus color composite of Iba1+ α7nAChR + HMGB1 + DAPI

To test whether UCO activate vagal brainstem nuclei, we stained for c-Fos induction in the vagal dorsal motor nucleus of UCO group compared to control animals. This methodology had not yet been established in fetal sheep model with the currently available c-Fos antibody. Consequently, first, we confirmed the specificity of this antibody for ovine brain tissue and the presence of the receptor in the fetal near-term ovine brainstem, cerebellum, and cortex (Additional file 2: Figure S1A and B). Both 62 kDa and 55 kDa c-Fos proteins were detected as expected. However, we were unable to establish reliable staining of this activation marker in our paraffin-embedded tissues (Additional file 2: Figure S1C). Therefore, our findings regarding possible vagal brainstem activation (or the absence thereof) are inconclusive at this stage of investigation.

## Discussion

We demonstrate that UCO-induced insult simulating human labor results in increasing fHRV properties known to reflect fluctuations of vagal activity. These fHRV properties are correlated with systemic and brain inflammatory responses and with shifts in nucleus-cytosol HMGB1 distribution in α7AChRs-positive microglia. We propose that this behavior is due to an increase in afferent CAP activity exerting anti-neuroinflammatory effects via microglial α7AChRs by limiting microglial activation. We observe that such effects occur in a brain region-dependent manner.

### Efferent effects of fetal CAP and its relation to HRV

Our findings support the first hypothesis that near-term asphyxia with worsening acidemia leads to vagal activation correlated to the degree of systemic inflammatory response, which suggests CAP activation [4, 16, 17]. The inhibition of brain regional cellular innate immune response was found in fetuses that were subject to acute worsening acidemia, but not to chronic intermittent hypoxia [17, 18]. Consequently, fetal acidemia, but not intermittent chronic hypoxia alone, is required for an aseptic induction of fetal inflammatory response and activation of CAP. Of note, duration of UCO insults, but not levels of lactate per se, correlated to IL-1β in the near-term fetuses [17]. In parallel with positive correlation of IL-1β and RMSSD, this suggests that chemoreceptor-mediated vagal activation results in part from the CAP-mediated sensing of the systemic inflammatory response that is more pronounced with longer duration of the stimulus.

### Afferent effects of fetal CAP on neuroinflammation via α7nAChR carrying microglia

To our knowledge, this is the first report relating systemic vagal activity in vivo and the cerebral inflammatory response in α7nAChR-labeled microglia, thus providing in situ and in vivo support for our second hypothesis, namely that CAP not only acts via efferent peripheral pathway to suppress systemic levels of inflammation but also targets neuroinflammation in the brain. This anti-inflammatory effect is probably achieved via the afferent branch of vagus nerve carrying cholinergic signaling to the brainstem where it is relayed to the higher subcortical and cortical regions. This notion is supported by several considerations. First, ~80 % of vagus nerve fibers are afferent and may provide feedback to the cerebral CAP system synchronizing its responses with those of the systemic CAP activity to the endogenous (e.g., acidemia as shown in the present study) or exogenous (e.g., bacteria or viruses) inflammatory stimuli [19]. Subdiaphragmatic vagatomy in adult rats activates microglia in the brainstem regions involved in vagus nerve signaling [20]. Second, vagus nerve stimulation (VNS) has been used to treat drug-refractory epilepsy for decades, yet the mechanism of action for VNS has remained elusive [21]. Anti-inflammatory effects of VNS may play a part in resetting the brain’s epileptogenic potential [21]. Third, recent nascent work on mapping the neuroimmunological homunculus has provided further evidence for the far-reaching connection between peripheral inflammation and cerebral perception and responses that are likely to include the vagus nerve as one of the main communication pathways both to and from the brain [22, 23]. Fourth, unlike its other cholinergic counterparts, the α7nAChR can be activated by a primary ligand other than acetylcholine, i.e., choline [24]. Hence, the α7nAChR can function in areas of the brain devoid of cholinergic transmission per se, where the far more ubiquitous choline may act as a substitute ligand. This may further contribute to a widespread effect of cholinergic signaling in the brain via the α7nAChR.

Furthermore, we demonstrate in situ α7nAChR expression in fetal sheep microglia and the modulation of this receptor’s immunofluorescent signal from nucleus and cytosol/membrane compartments by hypoxic acidemia and microglia status. We show that fetuses with higher RMSSD during severe acidemia showed lower MG activation in the WM. We further show that this may be mediated via α7nAChR in the WM’s activated MG. α7nAChR stimulation on adult and neonatal murine derived microglia in vitro reduces secretion of LPS-induced pro-inflammatory cytokine TNF-α [25, 26]. The CAP-mediated effect of afferent cholinergic activity on microglial HMGB1 release is further supported by negative correlation between the microglial thalamic cytosolic HMGB1 and vagal activation after reaching most severe acidosis. The α7nAChR expression is not static within a given brain region but rather appears to be dynamically regulated by stimuli such as hypoxic-ischemic injury which downregulates α7nAChR mRNA, unless the receptor is agonistically stimulated [7]. We propose that such stimulation may not only be exogenous, pharmacologically driven, but also endogenous and occurring via afferent CAP. Indeed, fetal cerebral inflammatory response and ensuing CAP activation resulted in a pronounced microglial α7nAChR intensity per area increase in the periventricular WM and reduced cytoplasmic HMGB1 intensity per area in the thalamus, which suggests that CAP homeostatically modulates microglia activity toward a neuroprotective phenotype via α7nAChR, similar teleologically to what has been proposed by Tracey et al. for systemic peripheral efferent effects of CAP [5].

We identified microglia status-dependent differences in HMGB1 translocation between cortical and subcortical brain regions. These differences seemed amplified by the UCO insult. UCOs are known to induce a redistribution of the regional cerebral blood flow (rCBF) toward subcortical structures [27]. In the hippocampal subregions, HMGB1 translocation was noted in the active microglia, while in the cortical layers 1–6 this was true for the quiescent microglia. This may be explained by a reportedly stronger rCBF increase in the hippocampus than in cortex under a similar UCO insult [27]. The relative rCBF increase in the hippocampus may also increase its exposure to the systemically circulating pro-inflammatory cytokines and lactic acid which upregulates microglia [28, 29]. We observe this as a stronger Iba1+ signal, larger microglial cell bodies and processes with close proximity to the neurons (cf. Fig. 4). This finding further contributes to the observation that the hippocampus is one of the brain regions most susceptible to hypoxic-ischemic insults [27]. Further studies are needed to better characterize the fetal microglial phenotypes involved in the UCO insult using morphological, morphometric, and more sophisticated biomarker approaches.

### Is there evidence that UCO activate brainstem vagal centers?

Labor contractions induce widespread c-Fos activation in the hypothalamic and thalamic nuclei, hippocampus, and cerebral cortex of pregnant rats that were subjected to umbilical cord occlusions [30]. This and other studies done in postnatal animals have in common that tissues were collected within 1–2 h post insult and cryosections were prepared for c-Fos immunohistochemistry [31–33]. Our study differs in both respects: we collected tissues 24 h post insult and used paraffin embedding, since at the time the focus was on neuroinflammation and brain injury; c-Fos studies were not part of the plan. As such, albeit the herein reported c-Fos findings are not conclusive, we chose to provide the complete methodology for the scientific community to be able to continue our work using this solely available commercial antibody. At present, it remains possible that c-Fos activation due to UCO subsided when measured at 24 h post pH nadir or was not visible in the paraffin-embedded tissues and cryosections should have been used. Regardless, our conclusions on the potential role of CAP in the mediation of neuroinflammation stand and are subject to validation in future studies.

The analysis of positive controls in fetal brains that were exposed to afferent vagus nerve stimulation, a unique experimental model of vagus nerve manipulation established recently in our laboratory, showed yet again no clear presence of c-Fos immunostaining (Additional file 2: Figure S1C). Since the slides used for this validation were also paraffin-embedded, a standard procedure for our studies, we suggest that the likely conclusion to this aspect of the study is that cryosections are key to preserving and visualizing c-Fos, at least using this particular antibody.

In summary, we cannot, at present, draw any definitive conclusion that at 24 h post UCO no c-Fos signal is seen in the vagal dorsal motor nucleus. Cryosectioning is needed to bring out the signal. Here, we present the immunohistochemical approach in fetal sheep brainstem and confirm the validity of the antibody chosen using Western Blot. We leave this aspect to further development in future studies.

### Clinical implications

In a series of experiments in chronically instrumented unanesthetized fetal sheep, an important model of human pregnancy, we showed that a fetal systemic and cerebral inflammatory response required the presence of severe hypoxic acidemia and was not induced by chronic hypoxia alone as might occur in the late gestation human fetus during labor or antenatally with growth restriction, respectively [34]. Since RMSSD, as a measure of vagal activity, increases in response to perinatal acidemia and inflammation [4, 35], we sought to determine the extent to which measures of systemic and cerebral inflammation in the fetus relate to RMSSD and indicate peripheral and cerebral CAP activity. We have induced fetal inflammation with hypoxic acidemia [36]. This stimulus activates systemic and cerebral innate immune responses and corresponds to a frequently observed spectrum of fetal heart rate distress patterns during human labor. Severe fetal acidemia (pH <7.00) at birth is observed in ~0.5–10 % of human births [37, 38]. Approximately 20 % of these babies will have neurologic sequelae including hypoxic-ischemic encephalopathy (HIE) and cerebral palsy [39–41]. Every fifth baby with cerebral palsy will have had an asphyxial event during birth, and every 10th, some degree of inflammatory exposure, with antenatal growth restriction and chronic hypoxia identified as another major contributing factor [42]. This is supported by animal research indicating that chronic hypoxia, prior to an acute asphyxia event, alters the interplay between the fetal neuroinflammatory and neuronal responses to the insult, potentially exacerbating the long-term effects of the exposure [28]. Overall, antenatal hypoxia and perinatal hypoxic acidemia are major contributors to perinatal brain injury resulting in increased risk for acute or life-long morbidity and mortality [43, 44]. Our findings suggest that an endogenous neuroinflammatory control mechanism, CAP, plays a neuroprotective role in etiology of early perinatal brain injury and that this mechanism may be monitored non-invasively using trans-abdominal fetal ECG technology. The latter conjecture is supported by a series of recent studies in fetal sheep and human labor cohorts [45–48].

## Conclusions

Our study provides first evidence of a neuroimmunological link in the late gestation fetus. Enhancing fetal CAP activity may suppress activation of microglia, therefore promoting neuroprotection. This may improve postnatal short- and long-term health outcomes through decreasing lasting brain injury.

## Methods

### Ethics statement

This study was carried out in strict accordance with the recommendations in the Guide for the Care and Use of Laboratory Animals of the National Institutes of Health. The protocol has been approved by the Committee on the Ethics of Animal Experiments of the University of Western Ontario (Permit Number 2006-091-08 “Intrapartum fetal monitoring: markers of hypoxic related injury”).

### Surgical preparation

Fetal sheep near term (0.86 gestation) of mixed breed were surgically instrumented with umbilical cord occluders to induce hypoxic acidemia (*n* = 10 + 5, further referred to as UCO group compared to control group which was also instrumented but not subjected to UCOs). The anesthetic and surgical procedures and postoperative care of the animals have been described [23]. Briefly, using sterile technique under general anesthesia (1 g thiopental sodium in solution intravenously (IV) for induction; Abbott Laboratories Ltd., Montreal, Canada; followed by 1 to 1.5 % halothane in O_2_ for maintenance), a midline incision was made in the lower abdominal wall, and the uterus was palpated to determine fetal number and position. The upper body of the fetus and proximal portion of the umbilical cord were exteriorized through an incision in the uterine wall. Polyvinyl catheters (Bolab, Lake Havasu City, AZ) were placed in the right and left brachiocephalic arteries, and the right brachiocephalic vein. Stainless steel electrodes were implanted biparietally on the dura for the recording of electrocortical activity (ECoG) and over the sternum for recording electrocardiogram (ECG). An inflatable silicone occluder cuff (OCHD16; In Vivo Metric, Healdsburg, CA) was positioned around the proximal portion of the umbilical cord and secured to the abdominal skin. Once the fetus was returned to the uterus, a catheter was placed in the amniotic fluid cavity and subsequently in the maternal femoral vein. Antibiotics were administered intra-operatively to the mother, (0.2 g trimethoprim and 1.2 g sulfadoxine, Schering Canada Inc., Pointe-Claire, Canada), fetus and amniotic cavity (1 million IU penicillin G sodium, Pharmaceutical Partners of Canada, Richmond Hill, Canada). Amniotic fluid lost during surgery was replaced with warm saline. The uterus and abdominal wall incisions were sutured in layers and catheters exteriorized through the maternal flank and secured to the back of the ewe in a plastic pouch.

Animals were allowed a 3–4-day postoperative period prior to experimentation, during which the antibiotic administration was continued. Arterial blood was sampled each day for evaluation of fetal condition and catheters were flushed with heparinized saline to maintain patency.

### Data and blood sample acquisition

A computerized data acquisition system was used to record pressures in the fetal brachiocephalic artery and amniotic cavity at 256 Hz, and the ECG and ECoG signals at 1000 Hz. All signals were monitored continuously throughout the experiment using Chart 5 for Windows (ADInstruments Pty Ltd, Bella Vista, Australia).

Fetal 3-mL blood samples were immediately spun at 4 °C (4 min, 4000 rpm; Beckman TJ-6, Fullerton, CA) and the plasma decanted and stored at −80 °C for subsequent cytokine analysis. In addition, to characterize baseline health status of the animals, fetal 1-mL blood samples were analyzed for blood gas values, pH, glucose, and lactate with an ABL-725 blood gas analyzer (Radiometer, Copenhagen, Denmark) with temperature corrected to 39 °C.

At the end point of each experiment as detailed below, the ewe and fetus were killed with an overdose of barbiturate (30 mg pentobarbital sodium, Fatal-Plus; Vortech Pharmaceuticals, Dearborn, MI) and a post mortem was carried out during which fetal gender and weight were determined, and the location and function of the umbilical cord occluder cuff were confirmed. The fetal brain was then perfusion-fixed with 500 mL of cold saline followed by 500 mL of 4 % paraformaldehyde and processed for histochemical analysis as reported [16, 18, 49].

### Experimental procedures

As reported [17, 50], animals were studied through a 1- to 2-h baseline period, an experimental period of repetitive UCO with worsening acidemia, and were then allowed to recover overnight. Five additional age-matched additional animals were used as controls for immunohistochemistry.

After the baseline period which began at ~8 a.m., repetitive UCO were performed with increasing frequency until severe fetal acidemia was detected (arterial pH <7.00), at which time the UCO were stopped. Complete UCO was induced by inflation of the occluder cuff with ~5 mL saline solution, the exact volumes having been determined by visual inspection and testing at the time of surgery for each animal. During the first hour, a mild UCO series was performed consisting of cord occlusion lasting for 1 min and repeating every 5 min. During the second hour, a moderate UCO series was performed consisting of cord occlusion for 1-min duration and repeating every 3 min. During the third hour, a severe UCO series was performed consisting of cord occlusion for 1 min duration, repeated every 2 min, and this series was continued until the targeted fetal arterial pH was attained. Following the mild as well as the moderate UCO series 10-min periods with no UCO were undertaken, during which fetal arterial blood was sampled and arterial blood pressure, ECOG, and ECG data were recorded in the absence of fetal heart rate decelerations. After attaining the targeted fetal arterial pH of <7.00 and stopping the repetitive UCO, animals were allowed to recover for ~24 h.

Fetal arterial blood samples were obtained during the baseline period (3 mL), at the end of the first UCO of each UCO series (1 mL), and ~5 min after each UCO series (3 mL). In addition, fetal arterial blood samples were obtained between UCO at ~20 and 40 min of the moderate and severe UCO series (1 mL), and at 1, 2, and 24 h of recovery (3 mL). At ~4 p.m. on day 2, the animals were killed as described above.

### Measurements of inflammatory responses

#### Systemic IL-1β and IL-6 secretion

An ELISA was used to analyze in duplicate the concentrations of IL-1β and IL-6 in fetal arterial and maternal venous plasma samples as reported [17]. IL-1β and IL-6 standards were purchased from the University of Melbourne, Centre of Animal Biotechnology, Melbourne, Australia. Mouse anti-ovine IL-1β (MAB 1001) and IL-6 (MAB 1004) antibodies and rabbit anti-ovine IL-1β (AB 1838) and IL-6 (AB 1889) polyclonal antibodies were purchased from Chemicon International, Temecula, CA. Separate 96-well plates were coated with mouse monoclonal ovine IL-1β or IL-6 antibody (1:200, in 0.1 M NaCO_3_, pH to 9.6) and incubated overnight at 4 °C. The following day, plates were washed three times with wash solution (1× PBS with 0.05 % Tween, pH to 7.4) to remove excess monoclonal antibody. Plates were then blocked with assay diluent (555213, BD OptEIA, BD Biosciences) at room temperature for 1 h. Wells were then rinsed three times with the wash solution followed by aliquoting standards (40,000 pg/mL to 156 pg/mL and blanks) and samples, and incubation on the shaker at room temperature for 2 h. Subsequently, wells were rinsed three times with washing solution and the appropriate rabbit anti-ovine polyclonal antibody (IL-1β or IL-6, 1:500) was added to each well and incubated on the shaker for 1 h. Following five more washes, HRP-donkey anti-rabbit IgG (AP182p, Chemicon International, 1:10,000) was added to each well and incubated on the shaker for 1 h. The wells were then washed seven times with wash solution to remove all unbound secondary antibody, followed by the addition and 30 min incubation with substrate solution (51-2606KC & 51-2607KC, BD Biosciences) in the dark. Stop solution (1 N H_2_SO_4_) was applied and each well was read using a spectrophotometer at 450 nm, with 575 nm wavelength correction.

### Measures of neuroinflammation: immunohistochemistry to assess microglia counts

These methods and findings have been reported [17]. The focus of this study was the putative relationship between the changes in microglia (MG) count in the white matter we had reported and the fHRV as proxy for CAP activity. Below we report the method as it was applied for all brain regions. Brain regions that were selected from each animal for analysis were taken from a coronal section of blocked cerebral hemisphere tissue at the level of the mammillary bodies and included the parasagittal and convexity cerebral gray matter, periventricular white matter, thalamus, CA1, dentate gyrus (DG), and the combined CA2 and CA3 regions of the hippocampus. Each of the parasagittal and convexity cerebral gray matter regions was further divided into subregions combining layers 1, 2, and 3 and layers 4, 5, and 6.

The presence of MG in brain tissue sections was determined as previously reported [17, 18]. Briefly, to reduce staining variability, all immunohistochemistry was performed on the same day with the same batch of antibody and solutions. Tissue sections were incubated with an anti-IBA1 rabbit polyclonal antibody (1:500, Wako Industries, Richmond, VA), a robust marker for sheep MG, followed by incubation with biotinylated goat anti-rabbit IgG, and subsequently with avidin-bioton-peroxidase complex (both Vectastain Elite; Vector Laboratories, Burlingame, CA). Positively bound antibody/peroxidase complex was detected using Cardassian Diaminobenzidine (DAB) Chromogen (Biocare Medical, Concord, CA).

Images were captured with a transmitted light microscope (Leica DMRB, Leica-Microsystems, Wetzler, Germany) at ×40 magnification. Six high-power field (HPF) photomicrographs (HPF area = 7 cm^2^) per brain region/subregion per animal were randomly collected as a 24-bit RGB color modeled image. The same illumination setting was applied to all images for all of the brain regions, and the microscope and lamp alignment were set up to provide even lighting with no background signal.

Positive MG cell immunostaining was quantified with an image analysis program (Image Pro Plus 6.0, Media Cybernetics, Silver Spring, MD). The image analysis system was first calibrated for the magnification settings that were used, then, using the Image Pro Plus’ RGB selection tool, color samplings of positive DAB stained areas were obtained from multiple brain regions as RGB histogram thresholds and tested for specificity against the negative control. Appropriate ranges of color were selected showing positive contiguous cytoplasmic staining as a criterion for MG cell count scoring, which were then applied uniformly to calibrated images for all brain regions. Scoring was then performed automatically by the software in a blinded fashion to experimental groups.

### Measures of neuroinflammation: immunohistochemistry to quantify HMGB1 translocation in Iba1+ microglia expressing α7 nAChR

Similar to MG analyses, whole brain coronal sections through the level of the mammillary body were selected for analysis. Paraffin sections (5 μM thick) were dewaxed and rehydrated through graded alcohols. All tissue sections for analysis were processed simultaneously, using pooled reagents and antibodies for consistency. Heat-induced epitope retrieval was performed in a vegetable steamer using sodium citrate buffer, pH 6.0, for 25 min, followed by slow cooling to room temperature. Sniper background blocker (Biocare Medical, Concord CA) was applied to reduce non-specific background. Subsequently, the antibodies described in Additional file 1: Table S1 were applied as follows. Phosphate-buffered saline (PBS pH 7.4) rinses were performed between each step and staining was performed at room temperature. A cocktail of Iba1 and α7 nAChR primary antibodies was applied and incubated for 1 h. A cocktail of Alexa 568 anti-Rabbit and Alexa 647 anti-Mouse secondary antibodies was applied and incubated for 30 min. Using a method exhaustively tested in our laboratory to utilize two antibodies from the same species, the ability of the anti-rabbit secondary antibody to bind further was then blocked with 4 μg/mL of pre-immune non-specific rabbit IgG for 30 min, before applying the conjugated Alexa 488 HMGB1 antibody and incubating for 1 h. Tissues were rinsed in PBS, counterstained with DAPI, and mounted in Prolong Gold anti-fade mounting media (Molecular Probes, Invitrogen, Carlsbad, CA) to preserve fluorescent signal throughout the image capture.

Images were captured on a Zeiss AxioImager Z1 microscope, equipped with an Apotome grid-a structured illumination device, which isolates optical slices much like a confocal microscope (Carl Zeiss Canada Ltd, Toronto, ON). For each animal, eight random fields of view in each brain region were collected for analysis. All images within each brain region were captured at the same instrument settings to ensure consistent illumination and detection parameters among samples. To avoid crosstalk between channels that might create non-specific intensity signals, bandpass dichroic filters for each dye were carefully selected based on the spectral profiles of the fluorescent tags and tested against controls that are positive for that desired dye wavelength, but negative for all other dyes used. Images were captured sequentially using one filter set at a time for each channel. The image analysis was done in Image Pro Plus 7.0 (Media Cybernetics, Bethesda, MD, USA) as published [51]. All image analyses were performed on 8-bit tiff images calibrated for scale measured per pixel on a scale of 0 (black)–255 (white) within the area of interest (cytosol or nuclei). Area was calibrated to square microns. Intensity/area yielded a mean intensity/square micron. In all analyses, samples of non-tissue background areas were measured for intensity, and positive signal histogram settings were chosen to selectively measure signal above this background. Thus, mean intensity/square micron area of the sampling region is reported for HMGB1 and α7 nAChR.

We co-labeled with the HMGB1, α7 nAChR, Iba1, and DAPI, yielding four separate channels that we used as follows (Fig. 4 demonstrates this four-channel approach including negative controls). The original four-channel images were exported as separate greyscale tiffs for each fluorescent channel, representing HMGB1 (Alexa488), α7 nAChR (Alexa647), Iba1 (Alexa568), and DAPI/nucleus. Using the DAPI and Iba1 channel images, we used binary masking techniques to isolate regions of interest (ROIs) specific to only the nuclear and cytosolic signal regions of MG whole cells in two distinct cell populations (further described below). Image Pro allows us to save these outlines as geographic regions and reapply them as counting regions to the other channel images. These outlines were saved and reapplied separately to the matching, isolated HMGB1, and then α7 nAChR greyscale images, and intensity and area data from within those nuclear and then cytosolic outlines was collected, yielding separate data points for nuclear HMGB1, cytosolic HMGB1, nuclear α7 nAChR, and cytosolic α7 nAChR.

We discriminated and report separately active and quiescent microglia (aMG, qMG) based on the morphological features of the Iba1+ cells and their location in relation to the neurons: as aMG were considered Iba1+ cells exhibiting large (~2× fold) soma and processes engulfing neighboring neurons, while qMG did not exhibit either of these features (Fig. 4). We recognize that such distinction, albeit based on general consensus regarding distinct morphological features of active (hypertrophy and increased processes) versus quiescent microglia, is also somewhat subjective, when it comes to the notion of co-localization to or engulfing of neurons. We return to this in the Discussion section.

For analyses of HMGB1 and α7 nAChR intensity per area between the brain regions within each group, we had to take into account that their absolute values could not be compared due to region-specific optimized acquisition as described above. For HMGB1, we worked around this limitation by deriving relative measures of HMGB1 intensity per area as cytosol/nucleus ratio for each brain region, as HMGB1 translocation indices (the higher the ratio, the more translocation occurs), thus making them comparable.

### Do umbilical cord occlusions result in vagal activation on the level of the brainstem’s vagal motor nucleus: immunohistochemistry for c-Fos

We aimed to detect UCO-triggered vagus nerve-mediated neuronal activation in the dorsal motor nucleus of the brainstem. We referred to the Michigan State University Brain Atlas to locate the nucleus in our brain stem slides (cf. Additional file 3). We used an immediate early gene detection approach deployed widely in other species, such as rodents, by performing c-Fos immunostaining with a c-Fos transcription factor monoclonal anti-human antibody (LifeSpan Biosciences, Seattle, WA) generated from a peptide corresponding to amino acids 128–152 of human c-Fos. This region has 100 % homology to the sheep c-Fos protein. This is the only commercially available antibody expected to generate signal in sheep tissue. We validated this antibody in our available ovine brain tissue homogenates using Western blot (described below, Additional file 2: Figure S1A and B).

Staining was performed using the anti-mouse ImmPress polymerized peroxidase reporter enzyme kit (Vector Laboratories, Burlingame, CA), followed by detection using DAB Chromogen (Biocare Medical, Concord, CA). The functional chemistry of kit reagents was verified using antibodies and tissues known to work in our laboratories, specifically mouse anti-human myelin basic protein (LifeSpan Biosciences, Seattle, WA) in guinea pig brain tissue prepared using similar fixation and processing conditions. As positive and negative tissue controls, brain tissues from a different fetal sheep study being performed in our laboratory were run concurrently with UCO and control animals. In this study, afferent (positive control) and efferent (negative control) bilateral cervical vagus nerve stimulation (VNS) was accompanied by intravenous injection of endotoxin to trigger inflammatory response in fetal sheep near term [45, 46, 52, 53]. Twelve dilutions of c-Fos antibody were tested with various antigen retrieval methods. Dilutions ranged from 1 μg/ml, which yielded no stain, to 20 μg/mL which yielded very high background in all cells regardless of antigen retrieval method. Several antigen retrieval methods were attempted and compared. Both 10 mM Citrate pH 6.0 heat retrieval and 10 mM Tris-HCL/1 mM EDTA/0.05 % Tween pH 9.0 nuclear antigen retrieval methods were attempted at 25 and 40 min time points, using both steam and pressure cooker methods. At 37 °C, 0.1 % Trypsin incubation was also tested at 15, 20, and 25 min. While trypsin at 20 min appeared to generate some nuclear signal in a few neurons in the afferent VNS animals at dilutions of 5–20 μg/ml, the signal was diffused with high levels of background stain and could not be found in the nuclei of neuronal cells in the motor nucleus of the vagus (Additional file 2: Figure S1C). Furthermore, this trypsin treatment damaged the tissues and caused them to become ragged and fall off the slides in places.

### Western blot for α7 nAChR and c-Fos

The presence of the α7 nAChR and c-Fos proteins in sheep brain tissue was analyzed by Western blot. Fetal sheep brainstem, cerebellum and cortex were homogenized in lysis buffer (1× RIPA buffer, Cell Signalling Technology, Cat # 9806) with fresh addition of PMSF (1 mM, Sigma P7626) just before use and quantified by BioRad Protein Assay method (BioRad Cat # 500-0006). At 120 V, 30 μg of protein was subjected to electrophoresis for 90 min in 10 % denaturing polyacrylamide gels. Proteins were then electrotransferred onto 0.45 μM nitrocellulose membrane (BioRad #162-0115) at 100 V for 2 h at 4 °C in transfer buffer (190 mM glycine, 25 mM Tris-base, and 20 % methanol, pH 8.3). After blocking for 1 h in TBST (0.5 % Tween 20, 50 mM Tris-HCl, 150 mM NaCl) with 5 % skimmed milk, blots were incubated with primary antibody (either a rat anti-chicken α7 nAChR monoclonal antibody (Sigma N8158) at 1:5000 dilution or a mouse anti-human c-Fos polyclonal antibody (LSBio, LS-C77393) at 1:500 dilution) in 0.5 % TBST with 5 % skimmed milk overnight at a cold room, followed by three washes (10 min each) with 0.1 % TBST. The blots were then incubated with secondary antibody (for α7 nAChR, a goat anti-rat IgG HRP (Life Technologies, #A18865) at 1:10000 dilution; for c-Fos, a rabbit anti-mouse IgG HRP antibody (Abcam, ab6728) at 1:5000 dilution) in 0.5 % TBST with 5 % skimmed milk for 1 h with agitation, followed by three washes (10 min each) with 0.1 % TBST. The blots were further incubated with ECL detection reagents (GE Healthcare #2209) for 3 min, and then imaged using ImageQuant LAS 4000 Mini system (GE Healthcare). Beta-actin was used as loading control; after striping and blocking, the blots were probed with a mouse anti-mouse β-actin monoclonal primary antibody (1: 500 dilution, Abcam, ab8226) and followed by a rabbit anti-mouse IgG HRP secondary antibody (1:5000 dilution, Abcam, ab6728) both in 0.5 % TBST with 3 % BSA, ECL detection, and imaging as described above. Precision Plus Protein Standards (BioRad 161-0374) was used to estimate molecular size of the target protein.

### Fetal heart rate variability analysis to gauge activity of the cholinergic anti-inflammatory pathway

The fHRV methodology was described elsewhere [2, 54]. Briefly, *R* peaks were triggered by steepest ascent criterion to derive fHRV, and RMSSD was calculated from 5 min of fHRV using Matlab 6.1, R13 (The MathWorks, Natick, Massachusetts, USA). We selected 10-min intervals of artifact-free fetal ECG for each time point. ECG-derived fHRV segments were analyzed time-matched to the cytokine samples taken at baseline and during the UCO series. In addition, fHRV measures were correlated to the brain’s white matter microglia cell counts and brain region- and cell compartment-specific α7nAChR and HMGB1 signals in the Iba1+ microglia (see above). CAP activation was measured as increases in fHRV measure RMSSD that reflects vagal modulation of fHRV [2].

### Statistical analyses

Blood gas, pH, IL-1β, and fHRV-derived measurements in response to repetitive cord occlusions were compared to the corresponding baseline values by one-way repeated measures ANOVA with Holm-Sidak method of correction for multiple comparisons. A generalized estimating equations (GEE) model was used to assess the effects of UCO on HMGB1 translocation while accounting for repeated measurements in space across the brain regions with AR [1] correlation matrix. We used a linear scale response model with animal group, MG type (qMG, aMG), and brain regions as predicting factors to assess their interactions using maximum likelihood estimate and Type III analysis with Wald Chi-square statistics. A similar analysis was made to assess the behavior of α7 nAChR intensity per area across the groups and MG type with HMGB1 translocation index as covariate, but using an independent correlation matrix (α7 nAChR intensity per area between brain regions within each group could not be compared, since absolute values had to be used; hence no repeated measurements across the brain regions were assessed for α7 nAChR intensity per area values). Correlation analysis was performed using Spearman correlation coefficient (IBM SPSS Statistics Version 21, IBM Corporation, Armonk, NY). Significance was assumed for *p* < 0.05. Results are provided as means ± SD or as median [55] percentile, as appropriate. Not all measurements were obtained for each animal studied (see Figure legends).

## Additional files

Additional file 1: Table S1. Immunohistochemistry reagents used to quantify HMGB1 translocation in Iba1+ microglia expressing α7 nAChR. **Table S2.** Effect of UCO, microglia status and brain regions on the HMGB1 translocation index. Parameter Estimates. **Table S3.** Effect of UCO, microglia status and HMGB1 translocation on α7 nAChR signal. (DOCX 82 kb)

Additional file 2: Figure S1. C-Fos in fetal sheep brain. A. Western blot establishing the specificity of this antibody in near-term fetal sheep brainstem, cerebellum and cortex. B. Western blot: raw data of the image shown in Fig. S1A. C. c-Fos immunohistochemistry (IHC) in near-term fetal sheep and guinea pig brainstems. *Top left*: positive control staining. The cervical vagus nerve trunks were stimulated bilaterally (the stimulation was applied proximal to the bilateral cervical vagatomy to ensure strictly afferent signaling). Note diffuse c-Fos signal with high levels of background stain. *Top right*: negative control staining. Similar procedure was performed as in afferent stimulation, except the stimulation was performed distal of the vagatomy site ensuring strictly efferent signaling. *Bottom left*: example of a UCO group fetal sheep staining. *Bottom right*: Here we demonstrate the IHC approach regarding secondary antibody and visualization techniques; as primary antibody we used MBP (details in Methods). (ZIP 85110 kb)

Additional file 3: Motor Nucleus of Vagus---Location. Methods supplementary material: Neuroanatomical approach to locating vagal motor nucleus in fetal sheep brain (PDF 1716 kb)
