# Supplementary material for: Decreased neuroinflammation correlates to higher vagus nerve activity fluctuations in near-term ovine fetuses: a case for the afferent cholinergic anti-inflammatory pathway?
Source: J Neuroinflammation. 2016 May 10;13:103. doi: 10.1186/s12974-016-0567-x (PMC4894374; doi:10.1186/s12974-016-0567-x)
Supplement: Supplementary file 1 — Immunohistochemistry reagents used to quantify HMGB1 translocation in Iba1+ microglia expressing α7 nAChR. Table S2. Effect of UCO, microglia status and brain regions on the HMGB1 translocation index. Parameter Estimates. Table S3. Effect of UCO, microglia status and HMGB1 translocation on α7 nAChR signal. (DOCX 82 kb) [file 12974_2016_567_MOESM1_ESM.docx]

**Tables**

**Table S1. Immunohistochemistry reagents used to quantify HMGB1 translocation in Iba1+ microglia expressing α7 nAChR**

| **Primary Antibody/Other** | **Company/ Catalogue#** | **Dilution** | **Secondary Antibody** | **Company** | **Colour** |
| --- | --- | --- | --- | --- | --- |
| α7 nAChR, Rat monoclonal | Abcam Inc, Cambridge MA  #019-19741 | 1:20 | Alexa 647 goat anti-Rat | Molecular Probes/Invitrogen, Carslbad CA | White |
| Iba1, Rabbit polyclonal | Wako Industries, Richmond VA  #N8158 | 1:250 | Alexa 568 goat anti-Rabbit | Molecular Probes/Invitrogen, Carslbad CA | Red |
| Preimmune Rabbit IgG | Vector Laboratories, Burlingame CA  #X0903 | 4ug/ml | NONE | --- |  |
| Alexa 488 conjugated HMGB1, Rabbit polyclonal | Novus Inc, Oakville, ON  #NB100-2322G | 1:50 | NONE  (Conjugated primary) | --- | Green |
| DAPI | Molecular Probes/Invitrogen, Carslbad CA  #D1306 | 300nM | NONE | --- | Blue |

| **Table S2. Effect of UCO, microglia status and brain regions on the HMGB1 translocation index. Parameter Estimates** | | | | | | | |  |
| --- | --- | --- | --- | --- | --- | --- | --- | --- |
| Parameter | B | Std. Error | 95% Wald Confidence Interval | | Hypothesis Test | | | |
|  |  |  | Lower | Upper | Wald Chi-Square | df | Sig. | |
| (Intercept) | .535 | .0646 | .408 | .661 | 68.416 | 1 | .000 | |
| [Group=UCO ] | .167 | .0793 | .012 | .322 | 4.445 | 1 | .035 | |
| [Group=Control ] | 0^a^ | . | . | . | . | . | . | |
| [Group=UCO ] * [Brain_region=CA1 ] * [Microglia_status=*active* ] | .073 | .0675 | -.059 | .205 | 1.173 | 1 | .279 | |
| [Group=UCO ] * [Brain_region=CA1 ] * [Microglia_status=quiescent ] | .090 | .0907 | -.088 | .268 | .989 | 1 | .320 | |
| **[Group=UCO ] * [Brain_region=CA3 ] * [Microglia_status=*active* ]** | .106 | .0536 | .001 | .211 | 3.931 | 1 | .047 | |
| [Group=UCO ] * [Brain_region=CA3 ] * [Microglia_status=quiescent ] | .019 | .0490 | -.077 | .115 | .149 | 1 | .699 | |
| **[Group=UCO ] * [Brain_region=DG ] * [Microglia_status=*active* ]** | .182 | .0445 | .095 | .270 | 16.795 | 1 | .000 | |
| [Group=UCO ] * [Brain_region=DG ] * [Microglia_status=quiescent ] | .102 | .0809 | -.057 | .260 | 1.589 | 1 | .207 | |
| [Group=UCO ] * [Brain_region=GM13 ] * [Microglia_status=*active* ] | -.102 | .0727 | -.244 | .041 | 1.951 | 1 | .163 | |
| **[Group=UCO ] * [Brain_region=GM13 ] * [Microglia_status=quiescent ]** | -.203 | .0712 | -.342 | -.063 | 8.112 | 1 | .004 | |
| [Group=UCO ] * [Brain_region=GM46 ] * [Microglia_status=*active* ] | -.062 | .0648 | -.189 | .065 | .902 | 1 | .342 | |
| **[Group=UCO ] * [Brain_region=GM46 ] * [Microglia_status=quiescent ]** | -.125 | .0593 | -.241 | -.009 | 4.451 | 1 | .035 | |
| [Group=UCO ] * [Brain_region=Thalamus] * [Microglia_status=*active* ] | -.051 | .0459 | -.141 | .039 | 1.219 | 1 | .270 | |
| [Group=UCO ] * [Brain_region=Thalamus] * [Microglia_status=quiescent ] | -.052 | .0553 | -.160 | .056 | .889 | 1 | .346 | |
| [Group=UCO ] * [Brain_region=WM ] * [Microglia_status=*active* ] | -.030 | .0234 | -.076 | .016 | 1.592 | 1 | .207 | |
| [Group=UCO ] * [Brain_region=WM ] * [Microglia_status=quiescent ] | 0^a^ | . | . | . | . | . | . | |
| **[Group=Control ] * [Brain_region=CA1 ] * [Microglia_status=*active* ]** | .117 | .0490 | .021 | .213 | 5.718 | 1 | .017 | |
| **[Group=Control ] * [Brain_region=CA1 ] * [Microglia_status=quiescent ]** | .254 | .0777 | .102 | .406 | 10.677 | 1 | .001 | |
| **[Group=Control ] * [Brain_region=CA3 ] * [Microglia_status=*active* ]** | .177 | .0811 | .018 | .336 | 4.755 | 1 | .029 | |
| **[Group=Control ] * [Brain_region=CA3 ] * [Microglia_status=quiescent ]** | .130 | .0584 | .015 | .244 | 4.933 | 1 | .026 | |
| **[Group=Control ] * [Brain_region=DG ] * [Microglia_status=*active* ]** | .118 | .0548 | .010 | .225 | 4.621 | 1 | .032 | |
| [Group=Control ] * [Brain_region=DG ] * [Microglia_status=quiescent ] | .057 | .0563 | -.053 | .168 | 1.037 | 1 | .309 | |
| [Group=Control ] * [Brain_region=GM13 ] * [Microglia_status=*active* ] | .088 | .0937 | -.095 | .272 | .891 | 1 | .345 | |
| [Group=Control ] * [Brain_region=GM13 ] * [Microglia_status=quiescent ] | .049 | .1170 | -.180 | .278 | .175 | 1 | .676 | |
| [Group=Control ] * [Brain_region=GM46 ] * [Microglia_status=*active* ] | .142 | .0932 | -.041 | .325 | 2.318 | 1 | .128 | |
| **[Group=Control ] * [Brain_region=GM46 ] * [Microglia_status=quiescent ]** | .228 | .0913 | .049 | .407 | 6.253 | 1 | .012 | |
| [Group=Control ] * [Brain_region=Thalamus] * [Microglia_status=*active* ] | .106 | .0704 | -.032 | .244 | 2.250 | 1 | .134 | |
| [Group=Control ] * [Brain_region=Thalamus] * [Microglia_status=quiescent ] | .112 | .1129 | -.110 | .333 | .975 | 1 | .323 | |
| [Group=Control ] * [Brain_region=WM ] * [Microglia_status=*active* ] | .001 | .0354 | -.069 | .070 | .001 | 1 | .981 | |
| [Group=Control ] * [Brain_region=WM ] * [Microglia_status=quiescent ] | 0^a^ | . | . | . | . | . | . | |
| (Scale) | .025 |  |  |  |  |  |  | |

| Dependent Variable: HMGB1_translocation  Model: (Intercept), Group, Group * Brain_region * Microglia_status (*active* or quiescent) |
| --- |
| a. Set to zero because this parameter is redundant.  GM13 and 46 are cortical grey matter layers 1-3 and 4-6, respectively; WM, white matter; CA1, CA3 and DG (dentate gyrus) are the hippocampal subregions  **Bold** entries are statistically significant results. |

**Table S3. Effect of UCO, microglia status and HMGB1 translocation on α7 nAChR signal.**

| **Parameter Estimates** | | | | | | | |
| --- | --- | --- | --- | --- | --- | --- | --- |
| Parameter | B | Std. Error | 95% Wald Confidence Interval | | Hypothesis Test | | |
|  |  |  | Lower | Upper | Wald Chi-Square | df | Sig. |
| (Intercept) | 15.224 | 5.1517 | 5.127 | 25.321 | 8.733 | 1 | .003 |
| [Group=UCO ] | 3.842 | 6.2720 | -8.451 | 16.135 | .375 | 1 | .540 |
| [Group=Control ] | 0^a^ | . | . | . | . | . | . |
| **[Group=UCO ] * [Microglia_status=*active* ] * HMGB1_trans** | -9.527 | 4.5915 | -18.527 | -.528 | 4.306 | 1 | .038 |
| **[Group=UCO ] * [Microglia_status=quiescent ] * HMGB1_trans** | -12.360 | 4.5838 | -21.344 | -3.376 | 7.271 | 1 | .007 |
| [Group=Control ] * [Microglia_status=*active* ] * HMGB1_trans | -3.069 | 8.2511 | -19.241 | 13.103 | .138 | 1 | .710 |
| [Group=Control ] * [Microglia_status=quiescent ] * HMGB1_trans | -3.264 | 7.9857 | -18.915 | 12.388 | .167 | 1 | .683 |
| (Scale) | 35.371 |  |  |  |  |  |  |

| Dependent Variable: alpha7nAChR  Model: (Intercept), Group, Group * Microglia_status (*active* or quiescent) * HMGB1_translocation |
| --- |
| a. Set to zero because this parameter is redundant.  **Bold** entries are statistically significant results. |
