# Supplementary figures and images for: Decreased neuroinflammation correlates to higher vagus nerve activity fluctuations in near-term ovine fetuses: a case for the afferent cholinergic anti-inflammatory pathway?
Source: J Neuroinflammation. 2016 May 10;13:103. doi: 10.1186/s12974-016-0567-x (PMC4894374; doi:10.1186/s12974-016-0567-x)

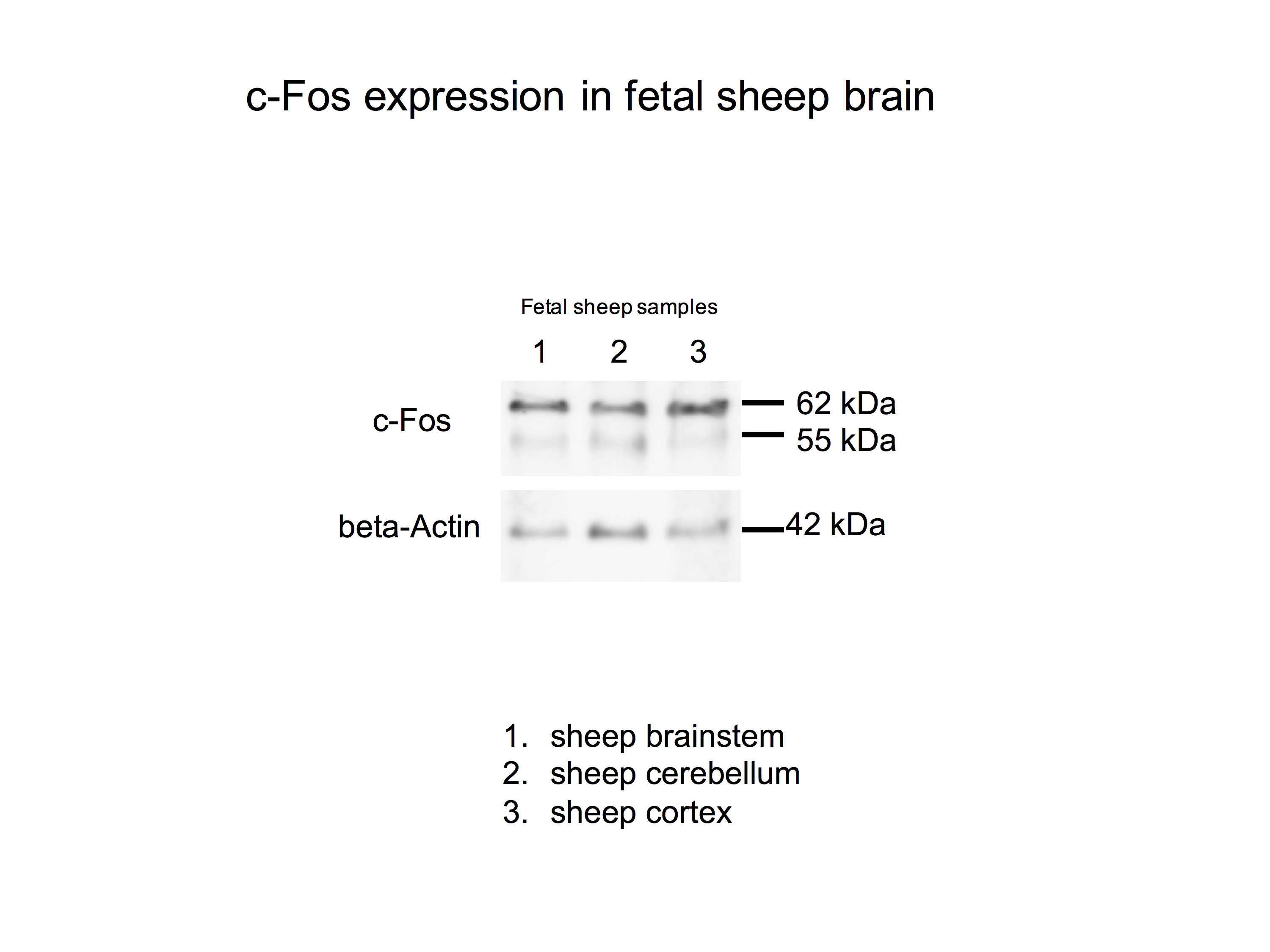

Supplement: Supplementary file 2 — C-Fos in fetal sheep brain. A. Western blot establishing the specificity of this antibody in near-term fetal sheep brainstem, cerebellum and cortex. B. Western blot: raw data of the image shown in Fig. S1A. C. c-Fos immunohistochemistry (IHC) in near-term fetal sheep and guinea pig brainstems. Top left: positive control staining. The cervical vagus nerve trunks were stimulated bilaterally (the stimulation was applied proximal to the bilateral cervical vagatomy to ensure strictly afferent signaling). Note diffuse c-Fos signal with high levels of background stain. Top right: negative control staining. Similar procedure was performed as in afferent stimulation, except the stimulation was performed distal of the vagatomy site ensuring strictly efferent signaling. Bottom left: example of a UCO group fetal sheep staining. Bottom right: Here we demonstrate the IHC approach regarding secondary antibody and visualization techniques; as primary antibody we used MBP (details in Methods). (ZIP 85110 kb) [file 12974_2016_567_MOESM2_ESM.zip › R2_c-Fos Western blot results-20151228-1 - FIG_S1a.tiff]

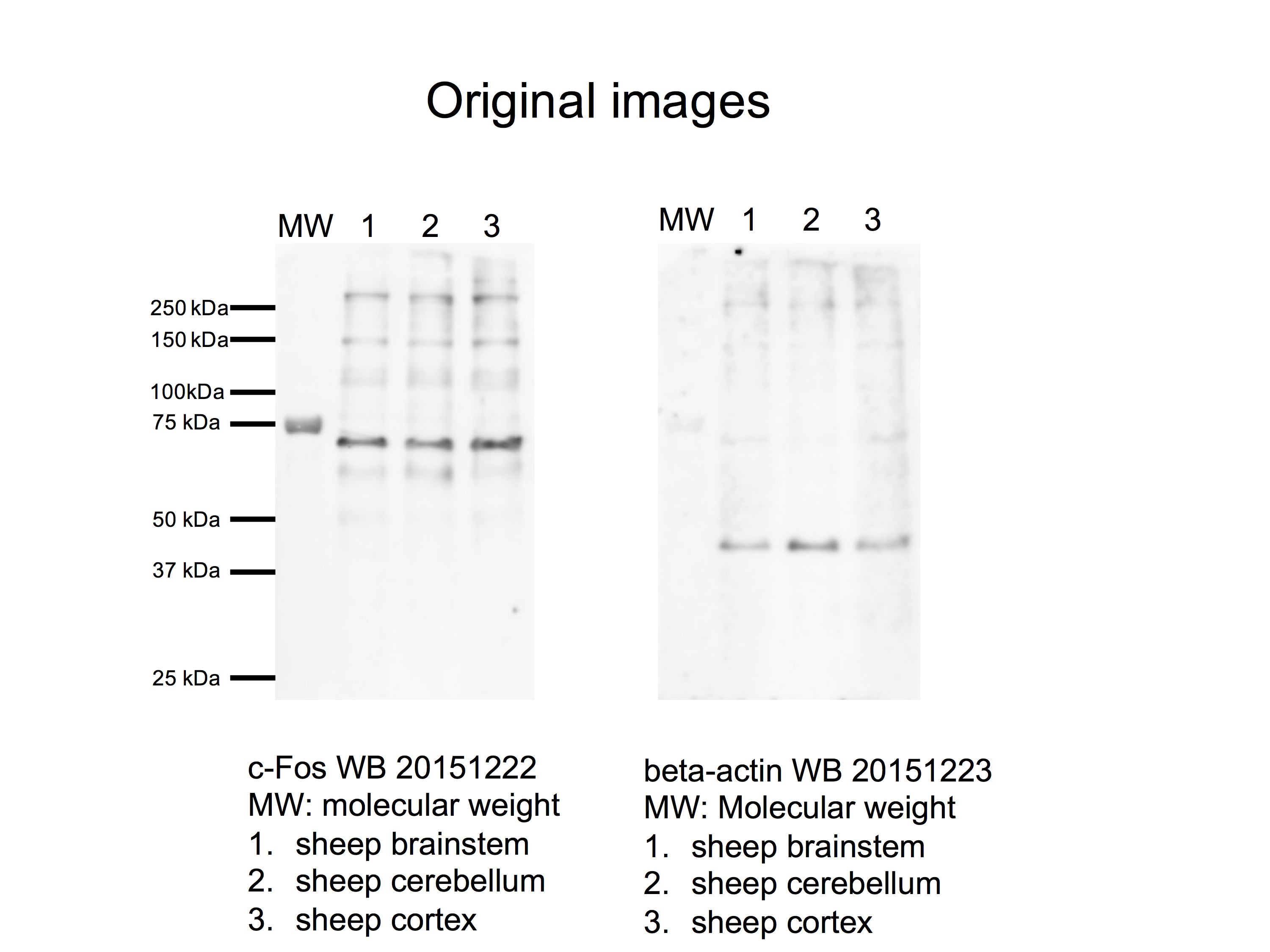

Supplement: Supplementary file 2 — C-Fos in fetal sheep brain. A. Western blot establishing the specificity of this antibody in near-term fetal sheep brainstem, cerebellum and cortex. B. Western blot: raw data of the image shown in Fig. S1A. C. c-Fos immunohistochemistry (IHC) in near-term fetal sheep and guinea pig brainstems. Top left: positive control staining. The cervical vagus nerve trunks were stimulated bilaterally (the stimulation was applied proximal to the bilateral cervical vagatomy to ensure strictly afferent signaling). Note diffuse c-Fos signal with high levels of background stain. Top right: negative control staining. Similar procedure was performed as in afferent stimulation, except the stimulation was performed distal of the vagatomy site ensuring strictly efferent signaling. Bottom left: example of a UCO group fetal sheep staining. Bottom right: Here we demonstrate the IHC approach regarding secondary antibody and visualization techniques; as primary antibody we used MBP (details in Methods). (ZIP 85110 kb) [file 12974_2016_567_MOESM2_ESM.zip › R2_c-Fos Western blot results-20151228-2 - RAW FIG_S1b.tiff]
