# Supplementary material for: Decreased neuroinflammation correlates to higher vagus nerve activity fluctuations in near-term ovine fetuses: a case for the afferent cholinergic anti-inflammatory pathway?
Source: J Neuroinflammation. 2016 May 10;13:103. doi: 10.1186/s12974-016-0567-x (PMC4894374; doi:10.1186/s12974-016-0567-x)
Supplement: Supplementary file 3 — Motor Nucleus of Vagus---Location. Methods supplementary material: Neuroanatomical approach to locating vagal motor nucleus in fetal sheep brain (PDF 1716 kb) [file 12974_2016_567_MOESM3_ESM.pdf]

Motor Nucleus of Vagus-Location

Michigan State Sheep Brain Atlas [1]

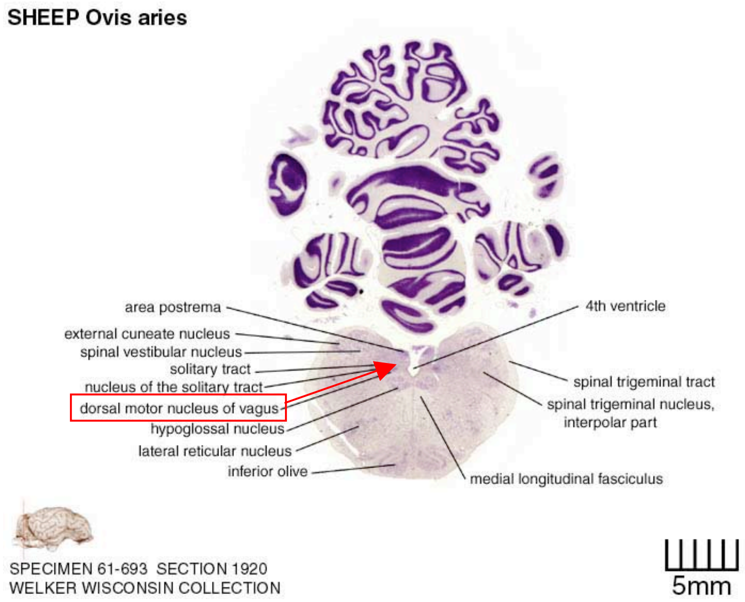

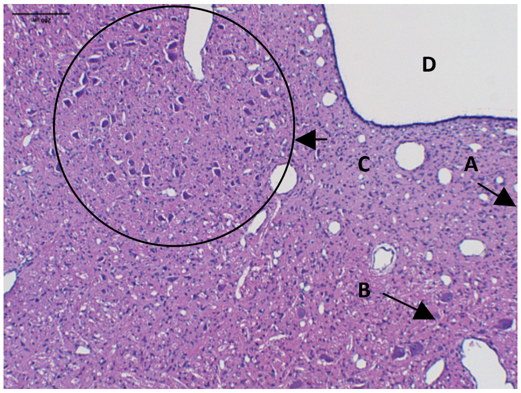

Low magnification H&E stain of brain stem from UCO #901 showing location of motor nucleus of vagus. A) midline of brain stem B) hypoglossal nucleus C) dorsal motor nucleus of vagus, D) 4th Ventricle (Scale Bar =200uM)

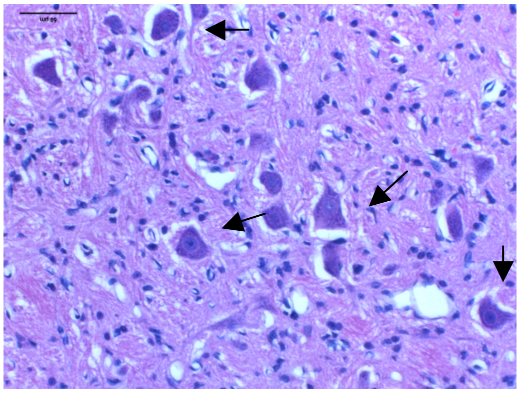

High magnification (20x) H&E stain of brain stem from UCO #901 showing detail of motor nucleus of vagus . Arrows indicate motor neurons cut through nuclei expected to show positive cFos signal following vagal stimulation. (Scale Bar =50uM)

1. https://www.msu.edu/user/brains/brains/sheep/scans/1920/image2.html
